# Pulmonary manifestations and clinical management of echinococcosis in a low-endemic region of Mexico: a 15-year retrospective cohort study at a tertiary hospital

**DOI:** 10.1186/s41182-025-00715-7

**Published:** 2025-03-10

**Authors:** Víctor Hugo Ahumada Topete, Misael Osmar Garcia Martin, Graciela Hernandez Silva, Alicia Jackeline Parra Vargas, David Martinez Briseño, Manuel Castillejos Lopez, Francisco Bernardo Perez Orozco, José Alberto Choreño Parra, Karina Danae Sevilla Gutiérrez, Elio Germán Recinos Carrera, Rosario Fernandez Plata, Anjarath Higuera Iglesias, Marco Villanueva Reza, Jolenny Jimenez Lopez, Arnoldo Aquino Gálvez, Luz María Torres Espindola, Joaquín Zúñiga Ramos

**Affiliations:** 1 Unidad de Epidemiología Hospitalaria e Infectología, Instituto Nacional de Enfermedades Respiratorias “Ismael Cosío Villegas”, Calzada de Tlalpan 4502, Col. Sección XVI, 14080 Ciudad de México, México; 2 Formación de Posgrado, Departamento de Enseñanza, Instituto Nacional de Endermedades Respiratorias “Ismael Cosío Villegas”, Calzada de Tlalpan 4502, 14080 Ciudad de México, México; 3 Departamento de Patología, Instituto Nacional de Enfermedades Respiratorias “Ismael Cosío Villegas”, Calzada de Tlalpan 4502, 14080 Ciudad de México, México; 4 Departamento de Imagenología, Instituto Nacional de Enfermedades Respiratorias “Ismael Cosío Villegas”, Calzada de Tlalpan 4502, 14080 Ciudad de México, México; 5 Laboratorio de Biología Molecular, Instituto Nacional de Enfermedades Respiratorias “Ismael Cosío Villegas”, Calzada de Tlalpan 4502, 14080 Ciudad de México, México; 6 Laboratorio de Farmacología, Instituto Nacional de Pediatría, Av. Insurgentes Sur 3700-Letra C, Coyoacán, 04530 Ciudad de México, México; 7 Dirección de Investigación, Instituto Nacional de Enfermedades Respiratorias “Ismael Cosío Villegas”, Calzada de Tlalpan 4502, 14080 Ciudad de México, México

**Keywords:** Echinococcosis, Pulmonary cyst, Zoonosis

## Abstract

**Background:**

Cystic echinococcosis has a low incidence even in endemic countries. It is a chronic and complex zoonosis that in many cases presents delay in diagnosis; it typically affects the liver in up to 90% of the cases, being disseminated pulmonary disease the most common in young subjects, while the rate of cases located only in the pulmonary parenchyma is low. In Mexico it is considered a disease of low endemicity.

**Material and methods:**

We retrospectively collected data from patients with suspected echinococcosis infection from the hospital discharge database.

**Results:**

Of the 70 patients in the database, 59 had a clinical history (84.3%), of whom 11 had a histopathological diagnosis of cystic echinococcosis and were included in this study, 67.6% were female, with a median age of 32 years (IQR 17–53.5). A total of 45.6% had some comorbidity, the most frequent being type II diabetes mellitus (80%); only 54.6% had lived in a rural area as a risk factor, while only 27.2% had exposure to canines. All cases were symptomatic, with a mean symptom duration of 49 days. A total of 81.8% had exclusive pulmonary disease, while the rest had simultaneous lung and liver involvement. No case presented spontaneous rupture. All cases received anthelmintic treatment and, in 9 cases, surgical resection of the pulmonary parenchyma. The only postsurgical complication was a chylothorax with adequate resolution. The median follow-up in months was 8.3 (IQR 3.7 to 10.7 months), and almost two-thirds of the cases presented dyspnea grade 2–3 (mMRC) as sequelae.

**Conclusion:**

Of all the patients studied with pulmonary echinococcosis, only two presented with hepatic-pulmonary hydatid disease, and spontaneous cyst rupture was not reported. About half had exposure to cattle as a risk factor, while no specific risk factor was identified in the rest of the subjects.

## Introduction

Human echinococcosis, caused by the tapeworm in larvae stage of the genus Echinococcus, is a zoonotic parasitic disease capable of affecting humans (accidental intermediate host) [27]. One of the principal species of cyclophyllid tapeworms of importance to the public health is *Echinococcus granulosus* which causes cystic echinococcosis (CE) (also known as hydatidosis). Its geographic distribution indicates a higher incidence in Western, Northern, and Eastern Europe, as well as in Central and Western Europe, the Middle East, and Central Asia [8], especially in sheep breeding areas, in South America, an annual average of about 5000 cases are reported, with a predominance in Argentina, Brazil, Chile, Peru, and Uruguay [31]. In North America, the oldest report dates to 1862, with a slow spread across the continent from Alaska to Canada and the Northern States of the USA. In Mexico, the first report occurred in 1962 [20], and since then, multiple isolated cases have been reported. Most of the case reports reported in Mexico—from the twenty-first century—occur in the hepatic parenchyma (confined) 14/16 cases (87.5%), while in the pulmonary parenchyma, there are two reports [32, 37]. The prevalence, according to the type of locality, was higher in rural areas (considered one of the most important risk factors) at 73.3%, while the remainder was found in highly urbanized areas. The primary risk factor reported was exposure to dogs, with some patients not having an identified risk factor [23, 32, 37].

Three possibilities have been proposed for Echinococcus in the larval stage not to lodge in the liver (organ mainly affected 80–90%), resulting in a primary pulmonary infection when it presents diameters < 0.3 mm and circulates beyond the hepatic sinusoids [38]; by lymphatic route, when the lymph drained by vessels of the small intestine mixes with the internal jugular vein passing through the heart thus reaching the lungs (bypassing the portal circulation) [22] and direct inhalation implanting into the lungs [34].

Therapeutic options are available: percutaneous PAIR (Puncture, Aspiration, Instillation, Reinfection; recommended for hepatic cysts); surgery (including enucleation, pericystectomy, cystostomy with capitonnage, open aspiration, segmental resection, and lobectomy [29],and pharmacological treatment only, which has shown effectiveness in cases of pulmonary hydatid disease that are irresectable [18]. In Mexico, 87.5% of the reported cases have undergone surgical management, primarily following the PAIR scheme (hepatic cases) [30, 37], followed only by antiparasitic treatment. For uncomplicated solid cysts that spontaneously become inactive (CE4–CE5 stages of the WHO-IWGE classification [9]) and remain stable throughout clinical follow-up, the recommended therapeutic option is the “Watch and Wait” approach, which consists of regular ultrasound monitoring without intervention on the cyst [26].

In this study, we aimed to describe the epidemiological characteristics and evaluate the pulmonary manifestations, imaging findings, and outcomes of a cohort of patients with cystic echinococcosis in an area of low endemicity.

## Materials and methods

### Study design and patients

A retrospective study was conducted at the Instituto Nacional de Enfermedades Respiratorias (INER) “Ismael Cosío Villegas”, a national reference center specializing in the management of lung and airway diseases, in Mexico City. We searched the hospital discharge database for patients with ICD-10 codes B67.1-0.5, K76.8, K76.9, and J85, who attended our hospital between January 2008 and December 2023. The diagnoses K76 and J85 were included in our search due to their association with non-specific liver and lung pathologies. Given this nonspecificity, we deemed it necessary to rule out the presence of echinococcosis.

Patients were included if they were hospitalized with confirmed cystic echinococcosis, defined as with or without symptoms related to cystic mass plus typical imaging lesions (Balikian P. Jirayr & Mudarris F. Faysal, n.d.; [13, 21]) and histopathology compatible or surgical specimen with infection by Echinococcus spp. The surgical samples were re-evaluated by two different pathologists.

**Fig. 1:**
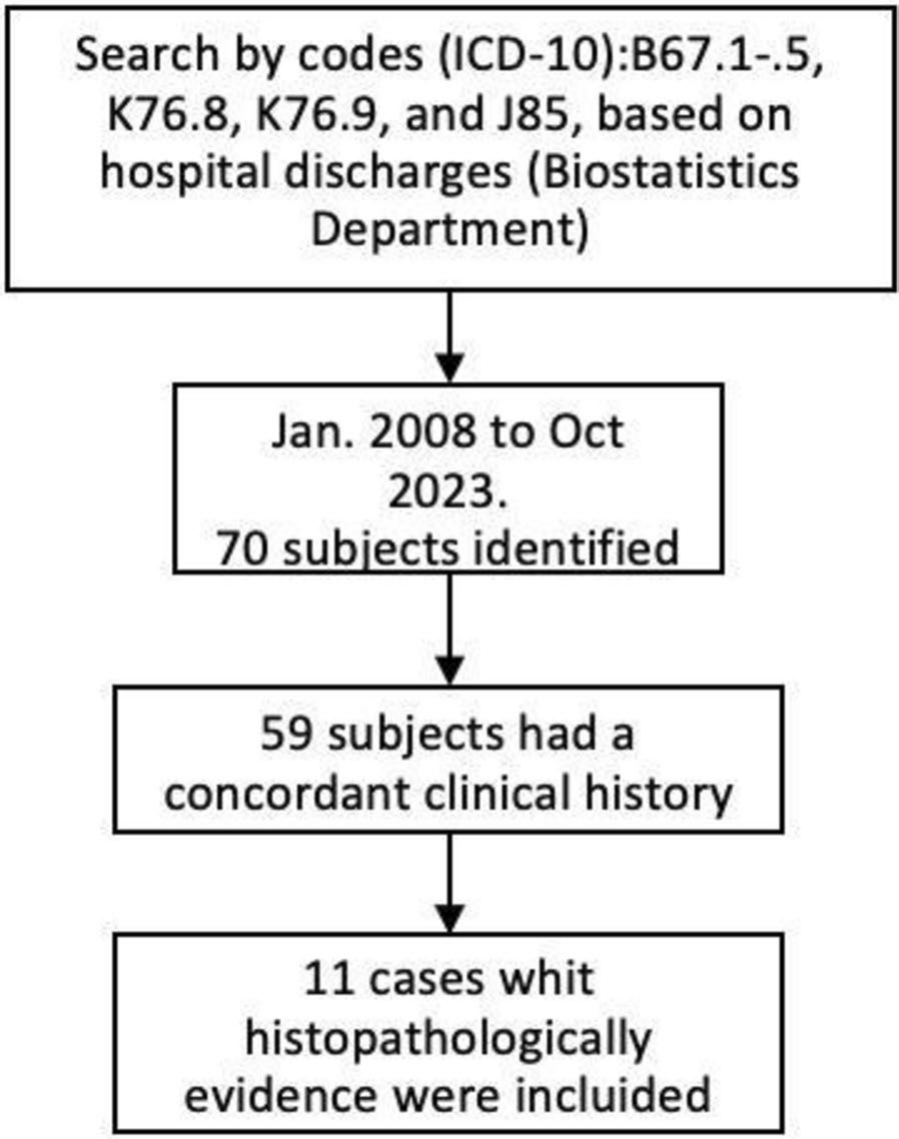
Recruitment algorithm. Diagram of the search, collection, and selection of cases

Data collected included demographics and epidemiological factors [free-ranging dogs, feeding dogs with viscera, home slaughter, slaughterhouses, dog ownership, living in rural areas and drink unboiled water [33]], clinical course, imaging findings, anthelmintic use, response to anthelmintic therapy, and outcome. Following the application of a structured assessment tool to standardize the collection of clinical information, we gathered the following data: start date of symptoms, symptoms present, date of hospital admission, date of tomography, imaging findings, date of hepatic ultrasound and findings, date of biopsy, date of surgery, type of approach, type of surgical resection and its indication, type of management received, start date of antiparasitic treatment and end date, type of antiparasitic, date of discharge, subsequent appointments, date of follow-up tomography, findings in follow-up tomography, start date of rehabilitation, residual symptoms and whether there were changes in them, date of last appointment, and details of the follow-up. The patients were selected according to the recruitment algorithm (Fig. 1).

### Statistical analysis

Data were captured and stored in a secure database before analysis. Data were summarized as mean, standard deviation, or frequency and percentage as appropriate. We calculate the hospital incidence rate per year using the formula: (number of diagnosed cases / total discharges) × 10,000. This formula was used as an indicator of the disease's incidence in the studied setting. All analyses were performed using SPSS version 21 (IBM Corp, Armonk, New York, USA).

## Results

Of the 70 patients in the database, 59 (84.3%) with a concordant clinical history (determined through the application of a structured clinical and paraclinical evaluation tool), of which 11 were histopathologically confirmed with echinococcosis and included in this study.

Clinical characteristics.

Of the affected subjects, seven were women and four men, with a median age of 32 years (from 8 to 81 years); nine cases were exclusively pulmonary, while two presented in liver and lung. Regarding the frequency of symptoms, eight presented with cough, seven with expectoration, and six with fever; only one patient presented with diarrhea (pulmonary echinococcosis) (Table 1). The median evolution of symptoms was 42 days until diagnosis (Insert Table 1).

**Table 1:** Clinical characteristics of study population

	Age/sex (year)	38/M (2008)	81/M (2010)	39/F (2010)	24/F (2013)	8/F (2015)	68/F (2017)	19/F (2019)	32/F (2022)	15/M (2022)	15/F (2023)	72/M (2023)	Frequency
													(% & *n*)
Symptoms	Localization	L/H	L	L	L	L	L	L	L	L	L	L/H	
	Fever		x		x			x	x	x		x	54.5 (6)
	Asthenia	x	x	x								x	36.3 (4)
	Adynamia	x	x	x								x	36.3 (4)
	Cough	x	x	x	x		x	x	x	x		x	81.1 (9)
	Expectoration	x	x	x	x			x	x	x		x	81.1 (9)
	Wasting syndrome	x	x	x						x		x	45.5 (5)
	Hemoptysis	x	x	x	x							x	45.4 (5)
	Pleuritic pain						x	x	x		x		36.3 (4)
	Dyspnea							x	x	x	x		36.3 (4)
	Abdominal pain	x	x	x		x							36.4 (4)
	Diarrhea									x			9.0 (1)
													Median (Q_25–_Q_75_)
Time to diagnosis	Days of symptoms until diagnosis	10	82	82	193	49	25	26	183	19	25	176	42 (22–177.7)

The frequency of symptoms presented with the median number of days of evolution of the symptoms presented until the moment of diagnosis. The sex (F: female, M: male) and age of each case are included

The hospital incidence rate is 3.17 cases per 10,000 discharges (Table 2).

**Table 2:** Hospital incidence rate

Diagnosis (year)	(1) 2008	(2) 2010	(1) 2013	(1) 2015	(1) 2017	(1) 2019	(2) 2022	(1) 2023	Median
Discharges per year	4444	4544	4273	4545	4739	4338	3938	4338	4394
Rate (per 10 000 discharges)	2.25	4.44	2.34	2.2	2.2	2.3	5.07	4.61	3.17

Hospital incidence rate of cystic echinococcosis. The table presents the number of discharges per year and the corresponding incidence rate per 10,000 discharges from 2008 to 2023. Median values for discharges and incidence rates are also provided

### Comorbidities and epidemiological characteristics

**Fig. 2:**
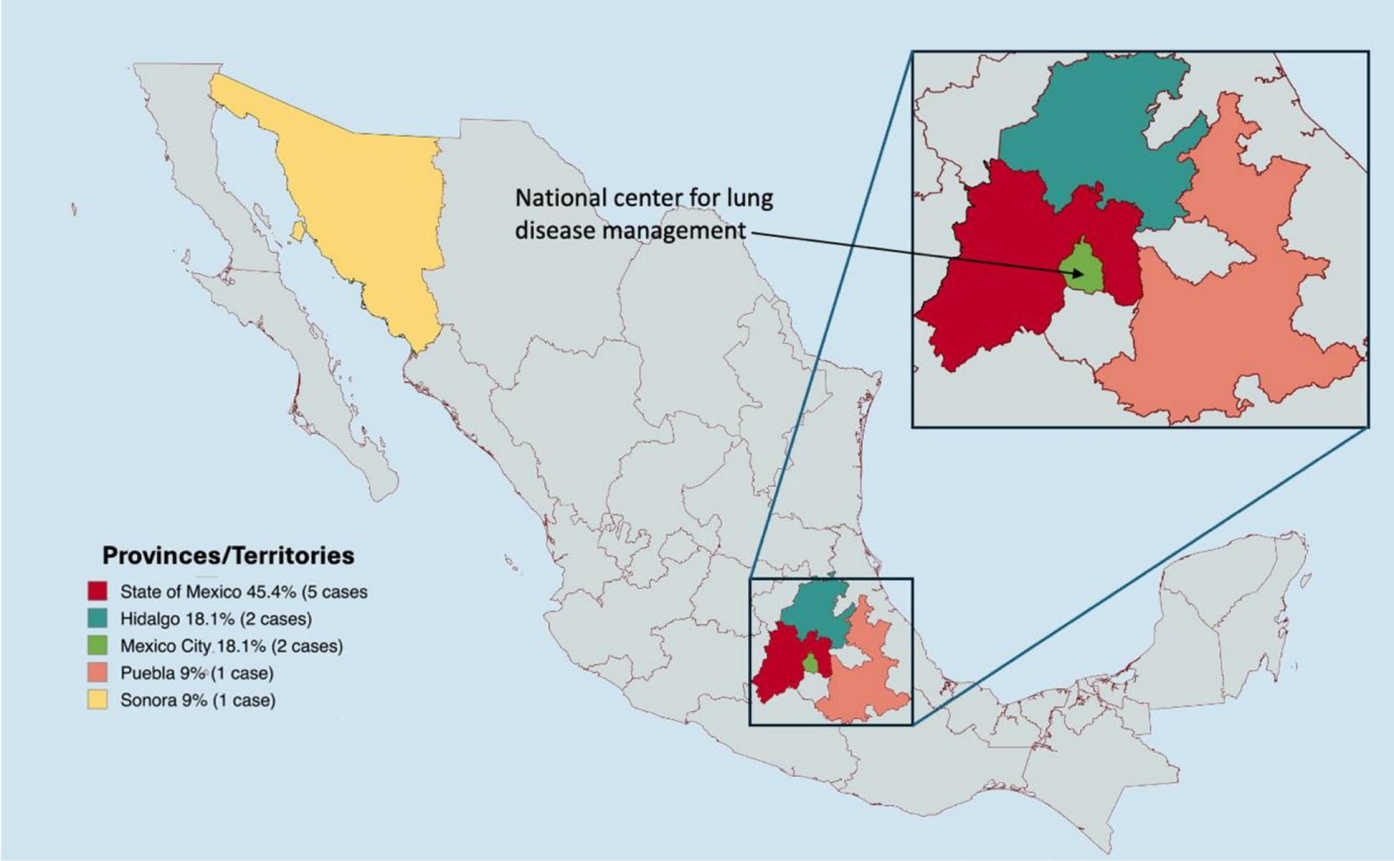
Map of the Mexican Republic where cases of echinococcosis and frequency by state are geographically located; the state of Mexico presented the highest incidence

At diagnosis, five subjects presented with comorbidities, with diabetes mellitus being the most common, affecting four cases. This was followed by arterial hypertension and malnutrition, each observed in three cases, the latter being associated with a consumptive syndrome. Among the epidemiological characteristics, the commonality for six subjects (6/11) was residing in a rural area, while five (5/11) reported exposure to livestock, which was typically kept in their backyards. Only three (3/11) subjects had dog ownership (living outside the house). The most frequent place of origin/residence was the State of Mexico, with 45.4%; the rest of the states presented frequencies of 1–2 subjects (Fig. 2).

### Imaging findings

All subjects underwent both plain and contrast-enhanced computed tomography (CT) scans, as well as hepatic ultrasounds. Nine subjects presented with pulmonary hydatid disease without involvement of additional organs (Table 3).

**Table 3:** Imaging findings of cases

Case	Tomographic manifestation	Cyst dimensions (mm)
2 (2008)	Liver abscess in segment V. Two hypodense cysts in segment two and lower right lung lobe	100.0 × 95.0 (hepatic)
1 (2010)	Multiple hypodense cysts. The largest is in the left pulmonary segment two, perilesional consolidation, and left upper lobe atelectasis, distortion of the pulmonary parenchyma architecture	48.5 × 60.0
3 (2010)	Single heterodense cyst in left lung segment 6	42.4 × 48.0
6 (2013)	Heterodense cyst in right lung segment 6 with camalote sign, air bubble, and pulmonary effusion	85.4 × 74.3
4 (2015)	Two hypodense cysts in the right lobe and middle lung lobes	60.6 × 67.2 and 54.4 × 41.8
5 (2017)	Ruptured cyst in lower left lung lobe with serpent sign and pulmonary effusion	28.1 × 31.7
8 (2019)	Hypodense cyst in right lower lung lobes	98.3 × 122.7
7 (2022)	Heterodense cyst in right upper lung lobe (inverted crescent sign) and perilesional consolidation	72.1 × 65.4
9 (2022)	Cyst in right lung segment 6 with a mass within a cavity sign	82.9 × 83.9
10 (2023)	Heterodense cyst in right lung segment 2 with a mass within a cavity sign and right hydropneumothorax	58.6 × 70.0
11 (2023)	Heterodense cyst in right lung segment two and anechoic hepatic cyst in segment VI	43.7 × 45.6 (lung) 30.3 × 24.0 (hepatic)

Description of the imaging findings using contrast-enhanced computed axial tomography in each case

**Fig. 3:**
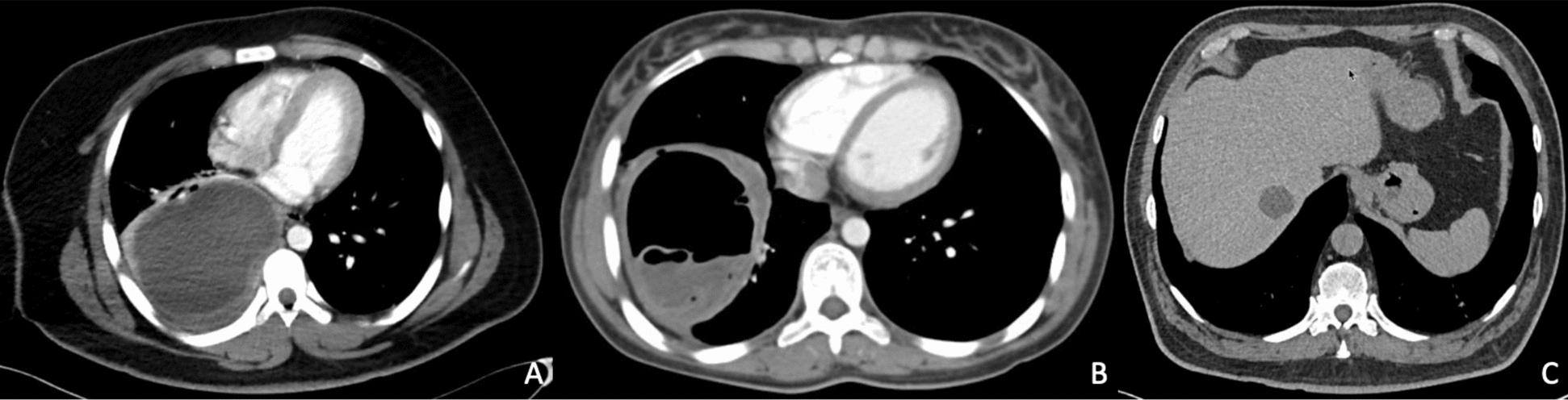
Cross-sectional contrast-enhanced CT scans of different cases. **A** Ruptured cyst is contained in the right lower pulmonary lobe with the characteristic crescent sign and the water lily sign (floating membranes due to the collapse of the endocyst). **B** Giant lung cyst (> 10 cm) uncomplicated. **C** Shows a single uncomplicated hepatic cyst in hepatic region VI

The right lung exhibited the highest frequency of involvement, affecting eight cases, with the lower lobe being the most affected; no bilateral involvement was noted (Fig. 3A, B).

Only one subject showed multiple cystic lesions disseminated in the left lung, accompanied by a pattern of pulmonary consolidation. Upon arrival, one patient experienced a cyst rupture as a result of a diagnostic surgical puncture. The most frequent concomitant findings included pleural effusion in three subjects, while atelectasis and hydropneumothorax were noted in different subjects. Hepatic hydatid disease (concurrent with pulmonary hydatid disease) without rupture was observed in two subjects: the first subject had two pulmonary cysts and hepatic disease located in segment V, while the second subject had one pulmonary cyst and hepatic disease in segment VI (Fig. 3C).

### Laboratory findings

Only one subject presented leukocytosis (23.8 × 10^3^/mm^3^) with neutrophilia (20.7 × 10^3^/mm^3^), while moderate eosinophilia only two cases (1.5 and 1.37 10^3/mm3 in localized disease and disseminated to the liver, respectively) with average total leukocyte count in two cases.

### Pathological findings

**Fig. 4:**
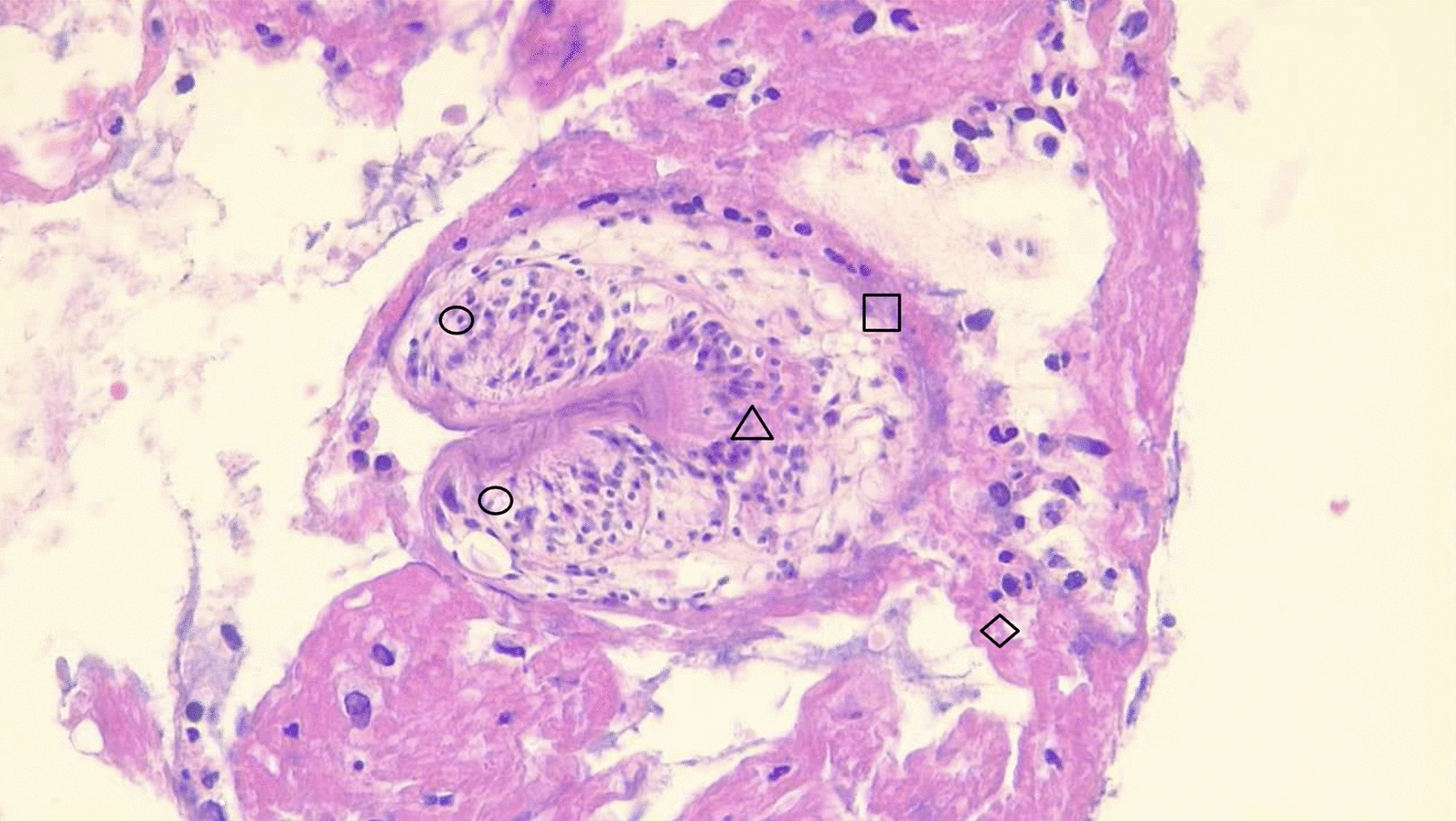
Photomicrograph of section of lung segment with hematoxylin and eosin stains observed with a 40 × magnification; you can see protoscolice ($\square$ ), crown radiata (△), suction cups (〇), pedicel (◇) with which it is attached to the germinative membrane

A total of ten histological specimens were analyzed (unfortunately, the surgical samples from one case were not available in the biobank of our institute). The cytological smears, stained with hematoxylin and eosin, revealed pathognomonic findings: a fibrous capsule, an acellular laminated membrane, a germinal layer containing multiple scolexes, and hooks displaying characteristic birefringence; these findings were observed in nine subjects (Fig. 4). Only one case exhibited extensive anthracosis, characterized by macrophages with abundant dark pigmentation and sinus hyperplasia while their macroscopic findings were cyst membrane and dense hyaline tissue with areas of anthracosis. While one of the surgical samples could not be re-evaluated (not available in the pathology bank), it was considered valid due to the information contained in the histopathological report and was documented as an “echinococcal cyst”.

### Surgical treatment

Of the nine patients with exclusive lung disease, surgical management was established as the cornerstone of treatment for seven. Lobectomy was performed in six of these patients, while segmentectomy was carried out in one patient (base on from the findings in situ during surgery). Only one case of hepatic-pulmonary involvement underwent surgical management for the giant hepatic cyst (100 × 95 mm), while the pulmonary cysts were treated exclusively with antiparasitic medications. The other case of hepatic and pulmonary hydatid disease was managed with pulmonary segmentectomy, and the treatment for the hepatic cyst consisted of exclusive medical management. The surgical team prioritized quality of life and survival in each case.

### Medical treatment

All subjects in our population received an anthelmintic either after surgical resection (with preoperative prophylaxis in one case, which was later maintained) or upon determining the irresectability of the cyst. Albendazole was prescribed for nine subjects, while the remaining patients received mebendazole, with a median treatment duration of 8.4 weeks for albendazole and four weeks for mebendazole. The treatment cycles consisted of 3 weeks of anthelmintic administration (calculated based on dose per kilogram of body weight) followed by one week without treatment. In cases of hepatic-pulmonary hydatid disease, where the hepatic cyst was drained, metronidazole was administered according the surgical findings and the criteria established by the surgical team.

### Follow-up and outcome

Patients with pleural effusion, atelectasis, and pneumothorax associated with the cyst were treated with the placement of an endopleural tube and resolved satisfactorily. The only postoperative complication encountered was a chylothorax in a 25-year-old female subject, which was resolved by ligation and placement of a thoracic conductor clipped. Nine of the patients underwent follow-up and pulmonary rehabilitation, with a median follow-up of period 9.6 months. Of these, five persisted with grade 2 dyspnea (MMRC), two with COPD, and one patient experienced ongoing surgical site neuralgia, while the remaining patients were reported as asymptomatic at their last visit. No subjects presented recurrence (new cystic lesions) of the disease, evaluated by CT scan at 6 and 12 months. Three subjects continue in follow-up and rehabilitation therapy.

## Discussion

In at least 45.4% of our cases, we were unable to identify a clear epidemiological characteristic, which contrasts with other Mexican cases where specific epidemiological characteristics are reported (Arturo Rodríguez-Leal et al., n.d.; [20, 32, 37]). The epidemiological characteristics most strongly associated with the acquisition of Echinococcus is living in a rural area [1, 23, 33], which was also the most prevalent epidemiological factor in our cohort, accounting for 54.6%. Another commonly reported reported factors in the literature is the presence of dogs as definitive hosts of *E. granulosus* (King & Yamashita, 1957) including free-ranging dogs, feeding dogs with viscera, and dog ownership; however, only 27.3% of our cohort exhibited this factor.

Uncontrolled diabetes mellitus is recognized as a factor that increases the risk of superinfection [12], however, this complication did not occur among our subjects with this medical history (27.2%). Concerning the geographical distribution of the cases, our results are consistent with those reported in studies of Mexican populations, where 30 to 50% occur in the State of Mexico, an area characterized by a combination of rural and urbanized zones with non-intensive livestock farming (backyard farming) [11]. A community epidemiological study conducted in a rural area of the State of Mexico (not Mexico City) demonstrated a prevalence of 0.15% cases of echinococcosis [19].

The median age of our subjects is consistent with that typically reported. The clinical manifestations of the disease are related to the location of the cyst or its rupture, and our results align with those identified by other studies (Fanne, 2006). It has been reported that up to 50% of giant cysts may rupture spontaneously (associated with giant cysts [25], however, in our cohort, 18.1% presented with giant cysts, of which only one ruptured as a result of a diagnostic puncture for suspected neoplastic disease.

Although the presence of multiple lung lesions is relatively frequent globally (approximately 30%) [10], we observed a lower frequency (9%). The concomitant pleural effusion observed in 27.2% of our subjects is relatively atypical compared to other studies [5, 14], where atelectasis would be the most frequent (approximately 17%). Simultaneous hepatic-pulmonary involvement is reported to occur between 8 and 11% [4, 16], which is lower than the 18.1% observed in our cohort. It is important to emphasize that the other cohorts are reported in countries with high endemicity, while our cohort is from a low-endemicity area. Notably, none of the hepatic-pulmonary cases presented gastrointestinal symptoms or manifestations of hepatic involvement.

In our study, 9% presented non-specific histopathology. Although histopathology with pathognomonic is an accessible method for the diagnosis of echinococcosis [9], it presents a specificity reaching an error rate of up to 15.4% [36],due to this, the diagnosis should be complemented with molecular biology techniques.

Regarding surgical treatment, we did not find significant differences in the approach performed by our hospital when compared with the guidelines [6, 28] according to the temporality of the diagnoses. In medical management, there is evidence of the superiority of albendazole vs. mebendazole with respect to outcome, disease recurrence, and survival,however, our study presents an insufficient number of subjects to evaluate therapeutic superiority.

The only postsurgical complication was a chylothorax, which resulted satisfactorily, showing an atypical condition as reported in the literature [35]

In our medium and long-term follow-up, no subject died, contrasting with other cohorts [3, 15] with higher mortality (5–10%) but on par with that reported in Mexican population (Dominguez Aguilar & Mexico, 2003; [37]). In this study, there was no relapse at 6 and 12 months, in agreement with current cohorts (highly associated with the duration of antiparasitic treatment) [24, 39].

No studies are identified that evaluate follow-up in lung capacity or clinical sequelae in surgically managed pulmonary cases, focusing on evaluating disease recurrence and immediate and mediated postsurgical complications. In this cohort, grade four dyspnea (MMRC) reported at the first follow-up visit (72.7%) progressed to grade two (MMRC) or complete remission in 75%, following a personalized pulmonary rehabilitation scheme, which is a noteworthy finding of the present study.

## Conclusions

Patients with pulmonary echinococcosis were studied, with only two presenting hepatic-pulmonary echinococcosis; notably, none experienced spontaneous cyst rupture. In our cohort, only 27.3% reported dog ownership. Furthermore, 75% were diagnosed within 6 months of symptom onset. Following pulmonary rehabilitation, a reduction and, in the best cases, remission of sequelae are expected during follow-up. Lastly, it is crucial to emphasize that early detection through imaging techniques is crucial for the prompt treatment of echinococcosis, which can significantly improve the prognosis of this disease.

## Data Availability

No datasets were generated or analysed during the current study.
